# Correlates and Health Issues among Older Korean Immigrants Living Alone in the United States: A Scoping Review

**DOI:** 10.3390/nursrep14030139

**Published:** 2024-07-31

**Authors:** Jung-Eun Kim, Sun-Ok Jung

**Affiliations:** 1 Mennonite College of Nursing, Illinois State University, 100 N University St., Normal, IL 61761, USA; jkim133@ilstu.edu; 2 College of Nursing, Ewha Womans University, 52 Ewhayeodae-gil, Seodaemun-gu, Seoul 03760, Republic of Korea

**Keywords:** Korean immigrants, living arrangements, scoping review, living alone, ethnic minority, vulnerability

## Abstract

Older Korean immigrants are one of the most understudied and marginalized Asian ethnic groups in the United States, despite their rapid population growth. Many older Korean immigrants encounter distinct challenges in assimilating into their new country as first-generation immigrants, including cultural conflict, language barriers, low economic status, and a lack of social support. These issues may be compounded for those who live alone, which is considered a negative factor in their mental and physical health. However, little is known about the correlates and health issues of older Korean immigrants living alone. This study’s objective was to explore correlates and health issues among older Korean immigrants living alone. Based on established scoping review methodology five databases, CINAHL, PubMed, MEDLINE, SocINDEX, and Health Source Nursing/Academic Edition, were used to find relevant studies. Twelve articles were reviewed, and four major themes were identified as correlates and health issues among older Korean immigrants living alone in the United States: depression, changed family relationships, social interactions, and factors on general health and well-being. The findings have significant implications for healthcare professionals for understanding the unique culture, situation, and physical and psychosocial vulnerability of older Korean immigrants living alone.

## 1. Introduction

### 1.1. Who Are Korean Immigrants?

Korean immigrants, who come predominantly from South Korea, constituted the tenth largest immigrant group in the United States in 2019 and ranked fifth among Asian immigrants [1]. Political, economic, and military relations between the United States and South Korea caused the population of Korean immigrants to increase by 2500%, from 11,000 in 1960 to 290,000 in 1980 [2].

According to the Pew Research Center [3], in 2019, the number of Korean immigrants in the United States was 1,908,000. About half of Korean immigrants settled in the following states: California (30%), New York (8%), and New Jersey (7%) [2]. Los Angeles County, Orange County, Bergen County, Queens County, and New Jersey, in that order, contained the greatest number of Korean immigrants.

In comparison to foreign-born and native-born populations, the educational attainment of Korean immigrants is exceptionally high [2]. Fifty-six percent of Korean immigrants aged 25 or older possessed a bachelor’s degree or higher in 2019, compared to 33% of the foreign-born and native-born populations, respectively.

In general, Korean immigrants have higher incomes than both foreign-born and U.S.-born individuals. The median income of Korean immigrant households in 2019 was approximately USD 72,000, whereas it was USD 66,000 for U.S.-born households and USD 64,000 for all immigrant households [2]. In contrast, the percentage of uninsured Korean immigrants was marginally higher than that of native-born Americans but roughly half as high as that of the total immigrant population [2].

### 1.2. Older Korean Immigrants

In general, Korean immigrants are older than both the foreign-born and native-born populations as a whole. In 2019, the median age of Korean immigrants was 49, whereas it was 46 for the foreign-born population as a whole and 37 for those born in the United States [1]. Compared with young Korean immigrants, older Korean immigrants are one of the vulnerable subgroups of Asian immigrants in the United States, despite being among the fastest growing [4]. Although Korean immigrants have higher incomes compared to foreign-born and native-born populations [2], poverty rates among older Korean immigrants are high. Older Korean immigrants reported living below the poverty line at a rate of approximately 22%, whereas other ethnic older immigrants reported poverty at a rate of 11% [5]. In the study conducted by Lee and Holm [6], more than half of the participants were dependent on Supplemental Security Income (SSI). This figure is significantly higher than the 30% of the general senior population that receives SSI benefits.

As first-generation immigrants, the majority of older Korean immigrants face unique obstacles to survival in the host country [7]. Older Korean immigrants who came to the United States at a later age may find it more challenging to master daily living skills like learning a new language, earning money, and adapting to the new culture [6]. Furthermore, a significant proportion (90%) of older Korean emigrants are monolingual (only Korean), and over 70% encounter difficulties comprehending medical terminology, despite the materials having been translated into Korean [8]. The language barrier is a well-known contributor to the health vulnerabilities of elderly Korean immigrants [9]. Additionally, it is important to observe that the rate of limited English proficiency or linguistic isolation was higher in the geographic regions with higher Korean densities [10].

### 1.3. Older Immigrants Living Alone in the United States

The majority of older Korean immigrants receive financial and physical assistance from their adult children [11]. For older Korean immigrants, family support and family networks continue to be vital and central [12]. Korean elders may gain security, a sense of worth, a sense of identity, as well as material and financial assistance, from their interactions with adult offspring [6]. On the other hand, although adult children continue to provide the majority of instrumental support for older Korean immigrants, a significant number of them aspire to lead independent lives and avoid being a burden on their children [6]. According to the study by Park et al. [7], 30.5% of the older Korean immigrants in the survey reported living alone.

In fact, the global rise in living alone occurs across the age range, and the increase in older people living alone as a trend is more pronounced in developed countries in the West and some parts of Asia [13]. Prolonged life expectancy is the primary demographic factor contributing to the increase in the rate of older people residing alone [14]. According to the Pew Research Center, around 27% of adults aged 60 and older in the United States were living alone in 2020. It is anticipated that this proportion will rise as the Baby Boomer generation approaches retirement age [15].

Many older people face difficulties such as lack of health insurance, limited access to transportation, and lack of healthcare resources, which prevent or limit access to needed healthcare services [16]. With the challenges that older people face in maintaining optimal health and well-being, living alone may be one of the negative factors in their mental and physical health. In particular, older Korean immigrants who resided alone reported a greater prevalence of chronic diseases, social isolation, and depression [17,18,19]. It is concerning that a significant percentage of older Korean immigrants reside alone, as this creates unique difficulties for individuals with linguistic barriers and hinders social integration [20].

### 1.4. Research Gaps and Objective

Living arrangements significantly impact the psychological health of older adults [21]. Individuals who live alone typically have a more limited network of relatives and receive less emotional and instrumental support than those living with others [18]. In other words, living alone implies a lack of social support from family and friends as well as isolation from neighbors and relatives. However, despite the vulnerability among older Korean immigrants living alone in the United States, little is known about the correlates and health issues among them. Thus, the objective of this study was to explore correlates and health issues among older Korean immigrants living alone. This study is important in that it may provide essential information for healthcare professionals about the vulnerability of older Korean immigrants living alone and the need for culturally sensitive health services in this minority ethnic population.

## 2. Methods

### 2.1. Overview

Scoping reviews are a form of evidence synthesis in which pertinent evidence that satisfies predetermined inclusion criteria pertaining to the subject, domain, setting, principle, or matter being examined is identified and mapped [22]. The objective of scoping reviews is also to identify research gaps as well as to delineate essential concepts and forms of evidence [23]. We used the methodological framework of Arksey and O’Malley [24] to conduct a scoping review. Arksey and O’Malley’s methodological framework includes five steps, including identifying research questions, identifying relevant studies, study selection, charting the data, and collating, summarizing, and reporting the results. The Preferred Reporting Items for Systematic Reviews and Meta-Analyses checklist was used to ensure that the scoping review includes all essential items [25].

### 2.2. Stage One: Identifying the Research Questions

The specific research question in this scoping review is “What related factors or correlates and health issues are found among older Korean immigrants living alone in the United States?”

### 2.3. Stage Two: Identifying Relevant Studies

To find relevant studies, we used five databases, including CINAHL, PubMed, MEDLINE, SocINDEX, and Health Source Nursing/Academic Edition. Different search keywords were used during the initial literature search process. The search terms selected were broad and determined to capture all relevant studies. The search key terms used the following keywords in various combinations: Korean immigrants; United States or America or USA or U.S; older adults or elderly or geriatric or geriatrics or aging or senior or seniors or older people or aged 65 or 65+. The publication year was limited between 2000 and 2024, and we included only academic journals.

### 2.4. Stage Three: Study Selection

**Figure 1:**
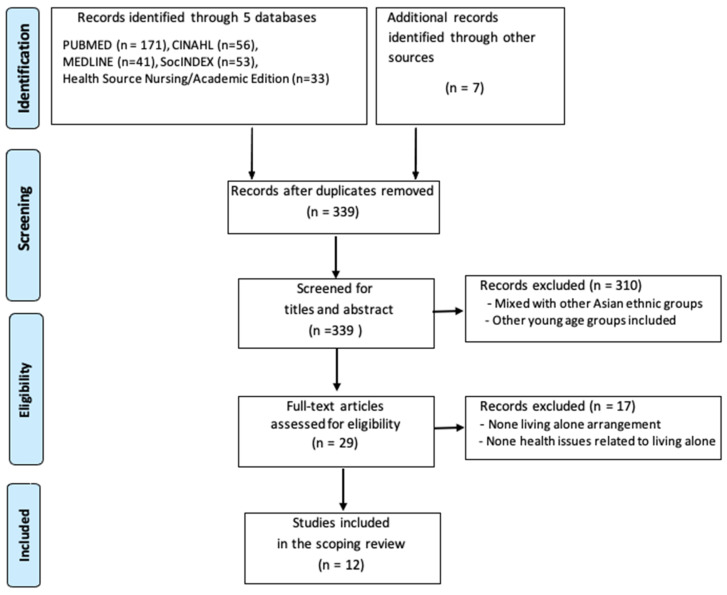
Flow diagram of study selection procedure.

A total of 361 studies were identified using the search terms. Through consensus agreement between two reviewers, 12 studies met the inclusion and exclusion criteria and were included in the scoping review. For manuscripts to be considered, they must be composed in the English language and specifically address older Korean immigrants living alone in the United States. We did not limit the journals’ publication areas. The title, abstract, research questions, and study results were examined by two independent reviewers in order to select the manuscripts. See Figure 1 for the flow diagram of the study selection process.

### 2.5. Stage Four: Charting the Data

Two reviewers used Mendeley to share the articles and consensus review. A spreadsheet was created to organize relevant information from the included studies. Data collected from each study included author names, publication year, study design, characteristics of participants, study location, and research results.

### 2.6. Stage Five: Collating, Summarizing, and Reporting the Results

In the final stage, the information was charted and sorted in accordance with the main themes and concerns raised in the literature review in order to facilitate synthesis and interpretation. Charts and thematic analyses were performed on the examined data. The data were analyzed by two researchers, and any analytical discrepancies were resolved through discussion until a consensus was reached.

## 3. Results

### 3.1. General Characteristics

Table 1 shows the general characteristics of the included studies. A total of 12 articles were selected to be reviewed with publication years from 2004 to 2022. Articles were published as follows: one in 2004, one in 2006, one in 2011, one in 2012, one in 2013, two in 2014, one in 2015, one in 2019, and three in 2022. Eleven studies (91.7%) were cross-sectional studies, and one study [26] was a randomized trial. All studies included older Korean immigrants with diverse living arrangements, such as living with a spouse, living with adult children, and living alone, but there was no study only targeting older Korean immigrants living alone among the 12 studies.

Among the 12 articles, seven studies (58.3%) were conducted in California, and three studies were in Baltimore–Washington metropolitan areas. The other two studies were conducted in Illinois (8.3%) and in multiple states including New York, Texas, Florida, and Hawaii (8.3%). The 12 studies reported the percentage of older Korean immigrants living alone in their sample: from 20% [26] to 70% [27]. The sample size was diverse, from 22 [28] to 2150 [7]. The total number of participants was highest in five articles (41.6%) with 100 to 300 people, followed by three articles (25%) with 100 or fewer people, and two articles (16.7%) with 1000 or more people. The number of older Korean immigrants living alone among the total participants was largest in four articles (33.3%) with 70 to 90, followed by three articles (25%) with fewer than 20, two articles (16.7%) with 20 to 50, one article (8.3%) with 235, and one article (8.3%) with 656. Ten studies reported the average age of the participants, from 70.5 to 76.3 years old. Two studies did not report the average age of the participants. Instead, they reported the age range of between 60 and 99 years old. The average age of the participants in seven papers (58.3%) was between 70 and 75, and in three papers (25%), it was over 75.

### 3.2. Thematic Analysis

As a result of the thematic analysis of the literature, four themes were derived. These themes include depression, changed family relationships, social interaction, and factors affecting general health and well-being. Since there were no articles only focusing on living alone, some results are discussed with other living arrangements. See Table 2 for the thematic analysis of the included studies.

#### 3.2.1. Depression

According to the studies [29,30,31,32], older Korean immigrants living alone were more likely to have higher rates of depression compared to those living with adult children or living with a spouse. Particularly, older Korean immigrants living alone exhibited the greatest incidence of mild (23.1%) and clinical (17.4%) depression [30]. Including all types of living arrangements, overall, the group with the least education level had the highest incidence of depression, while the group with the highest amount of education had the lowest incidence of depression [31]. This trend was the same in the study of Kim et al. [30] and Lee et al. [29], who observed a higher prevalence of depression among older Korean immigrants who had a lower level of education [29,30]. However, regarding the association between age and depression levels, there are mixed results. For older adults including all types of living arrangements, in comparison to the younger age group (those aged 60–69), the eldest age group (those older than 80) exhibited the highest prevalence of depression [31]. In contrast, Kim et al. [30] reported that the younger group showed higher rates of depression.

#### 3.2.2. Changed Family Relationships

In older Korean immigrants, the concept of family support has changed. Once regarded as authority figures in intergenerational families, older Korean immigrants feel they no longer hold a central position. A separation from their adult children was their preferred living arrangement [6,27]. The major reasons they prefer living separately from their adult children include privacy, freedom, and the desire to avoid burdening their adult children [6,27]. However, although older Korean immigrants want to live independently, they still rely on their children, particularly for English translation [27], financial problems, transportation, and emergency situations [6]. In the study by Wong et al. [27], a notable proportion of older Korean immigrants—70%—lived alone. These immigrants reported a heightened sense of familial significance and were more at ease seeking assistance from their offspring. As more older Korean immigrants want to live independently and broaden their social network, they need more help accessing government benefits and resources from ethnic community organizations and churches [27].

#### 3.2.3. Social Interactions

Friends comprised the majority of social interactions for older Korean immigrants, followed by in-person and telephone conversations with family members and relatives [26]. Even though they lived alone, older Korean immigrants living in senior group housing had ongoing interactions with neighbors in the same building and helped each other [26]. On the other hand, older Korean immigrants residing in single-family homes, townhouses, or apartments with adult children experienced greater social isolation and had fewer interactions with their peers [26]. In other words, regardless of the type of residence, the most important factor preventing social isolation for older Korean immigrants was the availability of social interaction with friends and neighbors. Through interactions with friends and neighbors, they shared health information such as medications or foods [26]. The living-alone group in the study of Han et al. [26] exhibited a higher rate of mental distress; this group was older, was more often female, had lower educational attainment, lower income, a greater number of chronic conditions, and greater functional disability, and experienced diminished family and community cohesion. In addition, older Korean immigrants living alone who are at higher risk of loneliness and social isolation were more likely to have poor nighttime sleep and poorer sleep quality [28,32].

#### 3.2.4. Factors on General Health and Well-Being

Based on the findings of Han et al. [26], older Korean immigrants who lived alone exhibited the following characteristics: higher proportions of females, older age, lower educational level, stayed longer in the United States, preference for senior group housing, and higher likelihood of Medicaid coverage. Additionally, they had higher levels of healthcare utilization and better blood pressure control [26]. In particular, women who lived alone showed better psychological and cognitive functioning [26]. According to Lee and Yoon [11], who studied low-income older Korean immigrants, there are no statistically significant associations between the variable “living alone” and the following outcomes: general health, anxiety, positive well-being, depression, self-control, or vitality. Instead, lack of English literacy and financial problems were associated with decreased positive well-being, while social support was related to increased positive well-being among older Korean immigrants [11,19]. Particularly, lack of English proficiency was a significant factor in anxiety, depression, lower self-control, lower vitality, lower general health, and decreased positive well-being [11]. According to Lee and Yoon [11] and Lee and Hwang [18], “living alone” itself was not significantly associated with general health. Instead, the level of social support, spiritual coping, lack of English proficiency, lack of transportation, and length of U.S. residency were more related to overall health status. Compared to older Korean immigrants living alone, living with someone and being employed for wages were significantly associated with better sleep quality [32].

## 4. Discussion

### 4.1. Principal Findings on the Four Themes

This study explored related factors and health issues among older Korean immigrants living alone in the United States using the previous literature. Due to the limited number of studies, the findings cover only specific health issues and correlates. The reviewed studies focus on depression, changed family relationships, social interactions, and correlates of overall health and well-being, which are the four major themes identified from the included literature.

The selected studies indicated that older Korean immigrants who resided alone were more likely to have elevated rates of depression than those who lived with adult offspring or a spouse. Depression is also related to several chronic conditions such as diabetes, arthritis, COPD, and digestive disorders [30]. Therefore, if older Korean immigrants living alone have depressive symptoms, they are more likely to face challenges in managing their chronic diseases. Therefore, policy support is needed to check the presence and degree of depression in advance and prevent it early. It is possible that a significant portion of older Korean immigrants remain depressed due to their limited English-speaking ability, resulting in limited activities and increasingly feeling more isolated [33,34]. We can also infer that low educational level may also be associated with low English-speaking ability. Therefore, appropriate education for older Korean immigrants living alone and customized programs to improve their English-speaking skills are needed. If they live alone and do not have family support, the living arrangement might impact their mental well-being negatively. Therefore, the provision of culturally appropriate healthcare providers and social welfare services to Korean seniors living alone is important.

According to the selected studies, older Korean immigrants experience changed family relationships. The majority of older Korean immigrants want to live independently and not be a burden to their adult children, but, at the same time, their children are still their major source of instrumental support. However, under the influence of a Westernized culture, the adult children of older Korean immigrants have altered the traditional Korean family values [35]. Adult children tend to live independently and value autonomy more than their old parents. Even though they live independently and do not want to rely on their children, it is a challenge for them. Immigration at a later age is a very stressful process for older Korean immigrants due to lack of language proficiency and cultural conflict. This results in inadequate treatment or a delayed diagnosis, both of which are associated with the common asymptomatic clinical presentation of chronic illnesses among Korean immigrants [35]. Older Korean immigrants had a higher risk rate of developing diabetes [30]; lower economic status and disparities in accessing health and social services were also observed [35]. As a result of their vulnerability, older Korean immigrants often depend on adult children for support. This situation engenders feelings of disrespect and a loss of identity for these immigrants as heads of the household and leaders [6]. To support independent living and maintain the well-being of older Korean immigrants, more culturally customized social resources are required. They can avoid a dependent role in the family and live more independently if they have improved access to healthcare services, transportation, financial support, and language barriers.

Social networks and interactions are crucial for older Korean immigrants who live alone in order to manage their chronic diseases and obtain vital health information. Particularly, friends are the primary source of social interaction for older Korean immigrants, as opposed to in-person or telephone relationships with family members or relatives. The living arrangement types, such as single homes, townhouses, or apartments, were not significant factors in their social interactions. Instead, their availability of social interactions with friends or neighbors was the most crucial part of preventing social isolation for older Korean immigrants. As mentioned in the study of Woo et al. [19], resources from ethnic community organizations and Korean churches could play a role as places to provide information and diverse activities as well as emotional support. To improve the social interactions among isolated older Korean immigrants living alone, culturally tailored family and community programs should be developed and provided. Since the current population of older Korean immigrants primarily comprises foreign-born first-generation immigrants, most have limited English proficiency. Language barriers and limited social networks outside of the country make older Korean immigrants socially isolated in their new society. However, this barrier can be mitigated by improving language support services and bilingual community programs, promoting improved social integration and overall health outcomes. Furthermore, fostering a sense of community support and belonging can be achieved through intergenerational programs that engage younger Korean Americans, which can help to bridge the cultural and language disparities.

The effects of living alone on health outcomes related to chronic diseases have not been examined much in previous studies, and there is a need for additional research in associating living alone status and health outcomes of chronic diseases. “Living alone” itself was not associated with general health and well-being for older Korean immigrants; instead, social support, language barriers, and financial problems had more of an effect on well-being. Particularly, lack of English proficiency was a significant factor in anxiety, depression, self-control, vitality, general health, and positive well-being. Living alone but also disengaging with others or eating alone were significantly correlated with loneliness among older Korean women immigrants [20]. Additionally, a longer length of residency in the United States for older Korean immigrants also was a significant correlate of increased anxiety, depression, and decrease of self-control, vitality, and general health [11]. In other words, older Korean immigrants living longer in the United States have more mental and physical distress than others who live for a shorter time in the United States [11]. However, this finding includes different living arrangements; thus, future studies need to examine the association between length of residency in the U.S. and general health and well-being while living alone.

### 4.2. Vulnerability of Older Korean Immigrants Living Alone

Despite a number of studies of older Korean immigrants, discussions of correlates and health issues focusing on older Korean immigrants living alone are lacking. Solitary living during old age can give rise to a multitude of difficulties, encompassing potential health risks, social isolation, and restricted availability of essential health services. A unique array of concerns must be made for older individuals who reside alone, including but not limited to finances, medication management, social engagement, transportation, housekeeping, and food [36]. As the Korean ethnic minority continues to increase in the United States, their unique vulnerability should be identified and explored. Depending on the type of living arrangements, the available healthcare resources and social support vary. As an ethnic minority immigrant group, older Korean immigrants are marginalized in accessing proper healthcare services, essential health information, education, and culturally customized community programs. Along with the natural aging process, older immigrants face more challenges in maintaining and improving their current health conditions related to cultural conflict, changed family roles, language barriers, and unstable economic status. In terms of the increase in the number of older people living alone, it is necessary to examine the correlates and health issues to develop effective healthcare services and programs for them.

### 4.3. Implications

To our knowledge, this study is the first scoping review focusing on older Korean immigrants living alone in the United States. Even though there are many studies about Korean immigrants, there is a lack of studies focusing only on older Korean immigrants living alone. This study found research gaps and suggested directions for future research targeting this ethnic minority immigrant group.

The findings of this study contain significant implications for healthcare professionals for understanding the unique culture, situation, and physical and psychosocial vulnerability of older Korean immigrants living alone. They are exposed to a higher prevalence of depression, ineffective health management, and limited access to healthcare services related to their language barrier, lack of social resources, and cultural differences.

### 4.4. Limitations

This scoping review makes a unique contribution to the literature pertaining to older Korean immigrants living alone in the United States. However, this study has several limitations. First, this study did not include literature review articles, theses, dissertations, or magazines. As a result, the number of articles that can be incorporated into the review may be constrained by the exclusion criteria. Due to the limited number of articles, the findings from this scoping review might provide a limited comprehensive view of the topic. Second, the reviewed articles included the states where many Koreans resided. Therefore, the findings of this study cannot represent older Korean immigrants living alone in other states that were not included. Third, due to the limited number of previous studies, this study includes a discussion of other living arrangements as well as living alone regarding some health issues and correlates. Therefore, we propose to conduct an in-depth study in the future targeting only older Korean immigrants living alone. Lastly, since the majority of the studies were cross-sectional, this review cannot provide trajectory changes or trends in the literature. Future studies are recommended to look at older Korean immigrants living in other states and use a longitudinal approach.

## 5. Conclusions

This study discovered that there is still a dearth of research on older Korean immigrants who live alone. Due to their status as an ethnic minority group, older Korean immigrants face marginalization and increased vulnerability. This investigation explored four overarching themes pertaining to health issues and correlated factors that had been the subject of prior research. In addition to the inherent process of aging, older immigrants face more obstacles when it comes to preserving and enhancing their health. In terms of the increase in the number of older people living alone, there is a need for additional research to investigate the health implications and correlates among older individuals living alone as well as to design healthcare programs and services that are patient-centered and culturally sensitive.

## Figures and Tables

**Table 1:** General characteristics of included studies (*N* = 12).

Variables	Categories	Frequency (%)
Publication year	2004~2006	2 (16.7%)
	2011~2013	3 (25.0%)
	2014~2016	3 (25.0%)
	2017~2019	1 (8.3%)
	2022	3 (25.0%)
Study design	Cross-sectional	11 (91.7%)
	Mixed methods study, A community-based cluster-randomized trial	1 (8.3%)
Region	California	7 (58.3%)
	Illinois	1 (8.3%)
	Maryland	2 (16.7%)
	Northern Virginia and Maryland	1 (8.3%)
	California, New York, Texas, Hawaii, Florida	1 (8.3%)
Number of Total participants (person)	<100	4 (33.3%)
	100~300	5 (41.7%)
	400~500	1 (8.3%)
	>1000	2 (16.7%)
Number of older Korean immigrants living alone from total participants (person)	<20	3 (25.0%)
	20~50	2 (16.7%)
	70~90	4 (33.3%)
	235	1 (8.3%)
	656	1 (8.3%)
	Not stated	1 (8.3%)
Mean age	70~<75	7 (58.3%)
	75~	3 (25.0%)
	Not stated	2 (16.7%)

**Table 2:** Thematic analysis of included studies (*N* = 12).

Themes	Discussed Contents in the Selected Studies
Depression	- A higher depression rate among older Korean immigrants living alone than those who lived with family members [29]. - No significant associations between living alone and depression [11] - Living alone had the highest prevalence of mild depression (23.1%) and clinical depression (17.4%) [30] - Older Korean immigrants who are less educated or living alone and do not engage in religious or social activities are more likely to experience depression [30] - Living alone (40%) is more prevalent than living with adult offspring (29.6%) and living with a spouse (14.4%) [31] - Limited English proficiency limits participation in various activities and increases feelings of isolation, which can lead to depression [31] - Living alone with lower levels of acculturation may exacerbate depression [32]
Family relationship	- Older Korean immigrants feel more central to the family, and are more comfortable asking children for help than older Chinese immigrants [27] - Older Korean immigrants choose to live alone to avoid conflict with adult children [27] - 22.5% of the participants wanted to live with their adult children [6] - 77.5% of the participants desired to live without their adult children. [6]
Social interaction	- No significant association between living arrangements and any of the psychosocial factors among older Korean immigrants [26] - Social isolation is not an inevitable consequence of living alone, especially for those without mobility impairments [7] - Lacking social resources is a more direct indicator of deficiency in social resources [7] - Living alone with lower levels of acculturation may contribute to social isolation and loneliness [32]
General health /well-being	- No significant associations between living alone and positive well-being, self-control, or vitality [11] - Lack of English literacy and financial problems were associated with lower positive well-being among older Korean immigrants [11] - Social support was related to positive well-being [11] - The presence of social networks outside the home might have positively influenced their blood pressure management [26] - Older Korean immigrants who lived alone had higher levels of healthcare utilization and better blood pressure control [26] - Older female Korean immigrants who lived alone showed better psychological and cognitive functioning [26] - Spiritual coping and social support showed significant positive correlations with general mental health [18] - Lack of transportation, lack of English proficiency, and longer length of residency in U.S. were negatively associated with general mental health [18] - “Living alone” itself was not associated with general mental health and general health perception [18] - Living alone is not associated with positive well-being [27] - Financial problems and lack of English proficiency were related to lower level of well-being being [19] - -Social support was associated with an increased level of well-being being [19] - Poor sleep quality was substantially correlated with living alone [32] - Unhealthy sleep patterns were highly prevalent among older Korean immigrants living alone [28]

## Data Availability

Since this study is a scoping review, there is no statistical data set.

## Public Involvement Statement

No public involvement in any aspect of this research.

## Guidelines and Standards Statement

This manuscript was drafted against the Preferred Reporting Items of Systematic Reviews and Meta-analyses guidelines for review research.
